# The practice and roles of condominium housing for tackling urban problems in the case of Gondar city, Ethiopia

**DOI:** 10.1016/j.heliyon.2022.e11957

**Published:** 2022-12-02

**Authors:** Bantayehu Ayalew Workineh

**Affiliations:** Department of Geography and Environmental Studies, Collage of Social Science and Humanity, University of Gondar, Gondar, Ethiopia

**Keywords:** Housing, Condominium, Urban problem, Ethiopia

## Abstract

Ethiopia today faces a contemporary urban problem of housing as a result of rapid population growth. The government designed the condominium housing program to afford houses for the residents, which requires the provision of infrastructure and faculties in the new developed areas. The purpose of this study is to demonstrate condominium housing institutional problems, actors, and roles in reducing urban problems. The researcher used mixed research approaches. The data was collected through key informant interviews, observation, satellite and aerial photo-image, and document reviews. As the data shows, local governments, municipalities, planning offices, small and micro enterprises, banks, contractors, and service providers participated in the condominium construction. However, there were poor administrations follow-up and resulted on the quality of the houses. The condominium can manage land; enhance housing supply, minimize service provision costs and expenses for compensation, reduce the number of expropriated farmers, minimize horizontal city expansion; and enhance relative housing affordability compared with plot-based houses.

## 1 Introduction

Housing is a basic human need (UN-Habitat, 2002), and urban development would not be functional without it. As evidenced by the global spread of poorly serviced, high-density informal settlements and slums, most cities in developing countries have failed to meet their people's housing needs. Housing demand is influenced by city economies, while housing supply flexibility is influenced by economic activity. The growth of the city's slums is closely linked to economic activity (Hammam & Sonia, 2014). In order to save the economy, land, and space in the cities, the best option is condominium housing rather than private apartments since it promotes cluster development (Schreiber, 1969).

Condominium housing comprises the simple ownership of a specific unit in a housing building as well as the shared ownership of supplementary space, utilities, and services (Mittelbach and Ebin, 1975; Rabenhorst and Ignatova, 2009). It has the capacity to change property practices and institutions, as well as the physical environment to accomplish effective land utilization and extensive economic wealth distribution (Schreiber, 1969). The condominium housing policy should promote low-income groups to purchase homes, as this would result in greater involvement in building and neighborhood upkeep, which would manifest itself in a superior quality of the residential environment (Ditkovsky and van Vliet, 1984; Schreiber, 1969; Harris, 2011); community participation (Maria Laura Ruiu, 2016); the ability to mortgage (Rabenhorst and Ignatova, 2009); and facilitate vertical subdivision (Harris, 2011). A condominium house is practicable when it is cost-efficient, egalitarian, environmentally viable, participatory, and legally and socially acceptable, unless it is not effective.

Condominium housing development in Ethiopia was intended to increase housing availability for the low-income population, decrease potential horizontal expansion, generate income for their families to help finance their homes, and improve the nation's production and distribution of resources (UN-Habitat, 2010). This has been accomplished through the collaboration of a variety of stakeholders, including financial institutions, government agencies, and brokers. Proclamation No. 370/2003 provides the legal framework for the program. Since 2004, a nationwide condominium construction program has been in place, with numerous houses constructed. However, in our country, the availability of plot based houses is significantly greater, indicating that, despite high demand, there may be misunderstandings and less attention regarding the condominium's role in addressing urban issues.

The previous research was conducted on condominium houses in Ethiopia and other countries. The researcher was interested in the management system, notably in Taiwan (Hung Ren Hsieh, 2009), organizational flaws in uncertain networks of governance (Yau, 2012, 2013); absence of collective management at the time of confronting interest (Yau, 2013, 2014); mechanism of housing management through public policy among low-income families (Marcuse, 1972; Frederick et al., 1999); and social and economic implications of condominium housing (Marcuse, 1972; Derrick et al., 1999; Mittelbach and Ebin, 1975). The other study looked at condominium management in Bahir Dar utilizing Ostrom's design principles, and found that the concepts were not adequately constructed and executed (Adamu, 2012). This study focuses on institutional administrative aspects, while these scholars are more concerned with management and socioeconomic repercussions. Furthermore, the study is evaluated by considering factors (aspects) that have not previously been considered by other researchers, such as land management, housing affordability as a function of cost, minimize service provision costs and expenses for compensation, and reduce the number of expropriated farmers.

The academician, policymaker, and housing expert would benefit from the study since it would demonstrate the role of condominiums in land management, housing supply augmentation, compensation cost reduction, and minimizing expropriation. It would also be a good lesson for countries that have experienced imbalanced demand and supply in housing, excessive compensation costs, and inadequate condominium house management. In general, scholars will use the study to conduct additional research, policymakers will use it to establish suitable policies and programs to address urban challenges, and housing specialists will use it to provide lessons on condominium housing implementation. As a result, the researcher aimed to demonstrate the condominium development programs' practice, and role in Gondar, Ethiopia.

## 2 Material and methods

### 2.1 Study area

**Figure 1:**
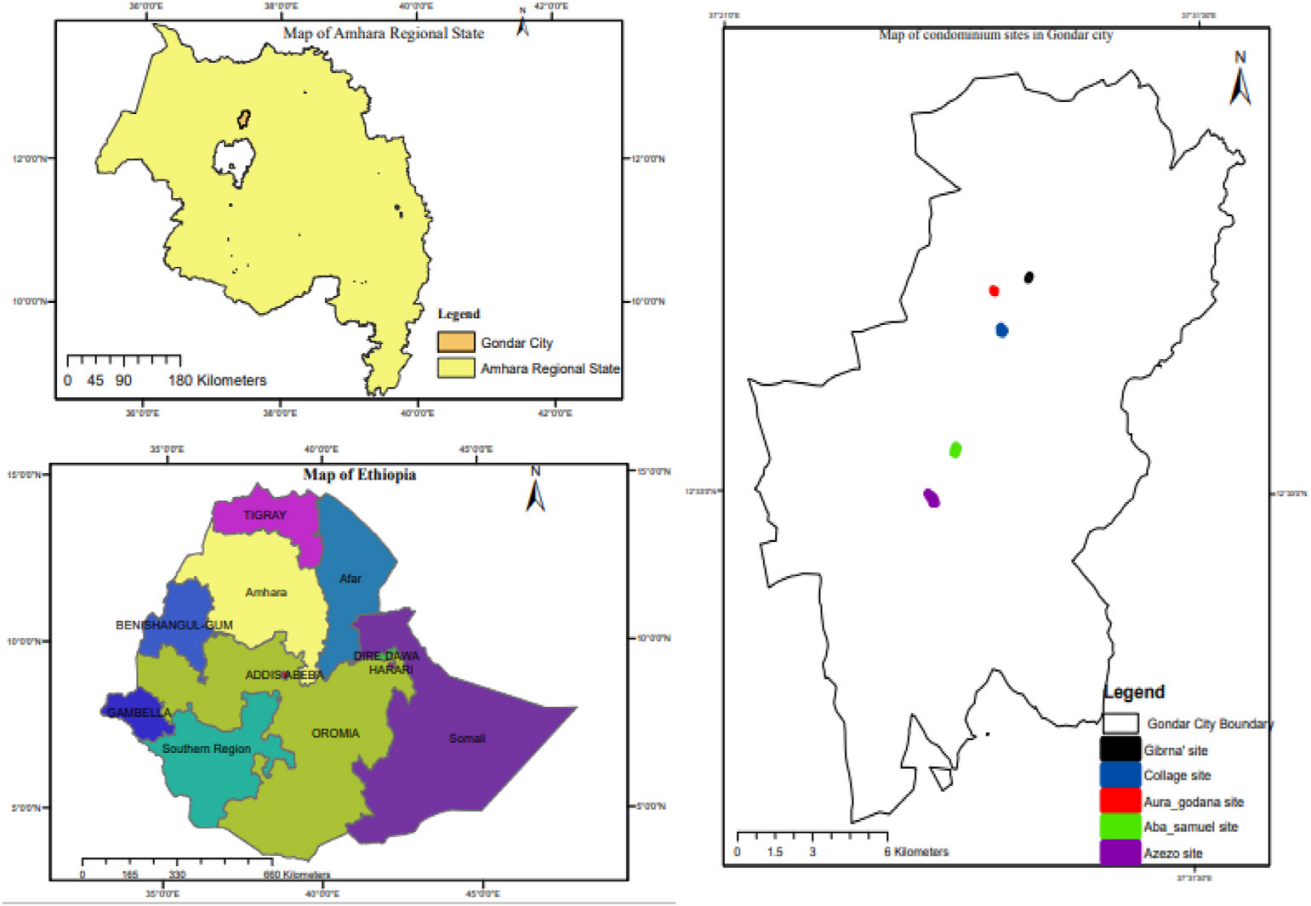
Map of the study area (source, Ethiopian mapping agency 2005: modified by author)

The investigation was carried out in Gondar, which is one of Ethiopia's historic cities. It was used in 1636 as the capital city. It is located at 12°45′ North and 37°45′ East. Sloppy land and man-made limits, such as the city being occupied by a historical region or heritage that hinders its expansion, are the primary natural constraints for the town's physical expansion. Figure 1 depicts the location of the research area.

The population of the city is rapidly rising. According to the Ethiopian Central Statistical Agency (CSA), the total population was 80,886, 112,249, and 207,044 in 1984, 1994, and 2007 (Central Statistical Agency, 1984, Central Statistical Agency, 2008, Central Statistics Agency, 1994). According to the World Population Review, there will be 395,138 people in 2022.

### 2.2 Methodology

#### 2.2.1 Research approach

The researcher used case study and quantitative research methods. The use of a case study allows for an in-depth examination of the complexity and distinctiveness of a certain initiative, policy, institution, or program from different angles (Simons, 2009). This research technique is adjustable and straightforward to incorporate many perspectives on the condominium's role and current state from inhabitants, officials, and documents. In addition, the case study approach incorporates a combination of interviews, personal observations, and documents from residents as well as official and office documents from the bank, housing, and planning agencies. Condominiums' importance was quantified using a quantitative technique.

#### 2.2.2 Sampling methods and sample size determination

When conducting research, sampling is the most important consideration, especially when the population is large. Purposeful sampling was employed in this study to obtain trustworthy and accurate information about the actors, financial, and administrative aspects of condominiums from the pertinent informants. The key informant samples were taken from housing development and administration offices, condominium association committees, the planning office, financial institutions, and the office of agriculture. The sample size is determined by the number of people who live in the area as well as their experiences and exposure to events. As a result, 25 people were chosen as primary informants from among 3346 condominium residents based on their experiences and involvement in the condominium in which they served as condominium committee members and lived for an extended period of time. The 20 officials were also chosen as key informants from relevant offices based on their experience and connections in condominium housing development and management. In addition, the report and regulation were selected based on their relevancy and attachment to the concept of the study. The following institutional office reports were chosen: Commercial Bank of Ethiopia Gondar branch, urban development and housing office, infrastructure provision office, central Gondar agricultural office, and CSA agricultural sample survey data. Also, regulation No. 01/2017, condominium proclamation No. 370/2003, and satellite photos of the entire condominium site in the study areas were used.

#### 2.2.3 Source and types of data

Primary and secondary data were used by the researcher. Through interviews and observations, primary data was gathered from officials, arena, condominium committees, and residents. Secondary data was gathered from office records, manuals, proclamations, and aerial photo photos.

#### 2.2.4 Data collection tools

The basic data collection instruments that have been used in the data collection procedure were key informant interviews, observation, satellite and aerial photo-images, and document reviews.

• Key informant interviews: they were used to collect data from the officials and leaders from selected offices to get detailed information in support and triangulate the secondary data.
• Observation: it is the collecting of data in the presence of the researcher and taking a photograph for analysis of the observed image.
• Satellite and Aerial Photo-image: This was done through the use of Arc GIS version 10.2 for determining the area of houses constructed and deciding the role of the condominium housing.
• Document Review: The reviews of relevant data, including reports, manuals, and proclamations, have been identified using this tool of data collection.

#### 2.2.5 Method of data analysis

After the collection of the data, the analysis of it comes next. The data was analysed qualitatively as well as quantitatively. The regulation-related documents, interviews, and observed data were analysed through thematic and content analysis. Whereas the data from the aerial image was analysed using Arc GIS version 10.2. This tool was used to identify the total area of the site by using an aerial photo and satellite images through measurement of the area, and it was used to estimate the amount of land saved. The data from the office report of financial, expropriation, land supply, and infrastructure minimized values, which were numerical in nature, were quantitatively analysed using formulated formulas and tables.

#### Ethical approval

The research was conducted in private with the permission of the departments of geography and environmental studies at the University of Gondar. With this permission, the data were collected from the offices and field observations using different techniques. The research is focused on an institutional program that was not primarily focused on people or animals. However, respected bodies involved in the study ensured their privacy and confidentiality from the time the data was collected until it was analyzed and published.

## 3 Findings and interpretation

This part of the research considers the findings and its interpretation emphasizes the practice, actors, and roles of condominium housing.

### 3.1 Practice of condominium houses

In this part, the legal frameworks, financial, supply and administration system of the condominium houses are discussed in detailed.

#### 3.1.1 Legal framework of the condominium house

Ethiopia has issued condominium proclamation No. 370/2003 with the goal of adopting various options beyond plot-based urban land use to minimize the gap between demand and supply of housing (Federal Democratic Republic of Ethiopia, 2003a). Furthermore, it aimed to preserve the urban area's beauty, improve urban land use and housing supply by allowing a large number of people to benefit from and collectively own a small piece of urban land, and create favourable conditions for private developers and co-operatives to purchase units, as well as all others who have a right in the condominium. The proclamation included the registration, certification, ownership, sale, and lease of a unit; expense and surplus; and the formation of the association, as well as the condominium's governance.

The condominiums were administered by the Construction and Housing Development Agency in the Amhara Regional States. However, the office was changed to the Construction Works Organization. As a result, the Amhara Regional State passed regulation No. 01/2017 for the management of condominium houses that were built under integrated housing development programs in 2007 and 2008 in major cities such as Gondar (Amhara Regional Urban Development Housing and Construction Office, 2017). The regulations sought to provide efficient condominium house management as well as a plethora of tasks and responsibilities for the government. The administration, the office's collaboration with financial institutions, the transfer of rights, the promotion of awareness, and the transaction of the houses are all important aspects of the organization. However, on the program's long-term viability, responsibility and specific tasks were not stated. As it was shown in the regulations, the management and maintenance of the condominium was run by the unit owner's association. Whereas most of the houses were rented, that reduced the fortification of the associations. For instance, at the Azezo condominium site, there were no unit owners' associations, and the condominium did not have a legal definition or set of regulations. As a consequence, there were conflicts between the owners and the tenants, the owners and the owners, the connection of the safety tanker with the drainage, and the non-standing of the common interest. However, there were associations at the other four condominium sites, but their bindings by rules were ineffective.

The following are some of the legal issues identified in the document and interviewed:

• The absence of a penalty for improper rule implementation and legal issues.
• Absence of supporting and controlling office: The condominiums were registered in the administration and security office but not in the condominium owners' association.
• The law is comprehensive, i.e., it does not incorporate what will be included in the administration and internal rules.
• House owners, renters, and association leaders have a low awareness of their duties and responsibilities.

In general, the regulations had strengths and weaknesses. Thus, the local government as well as other countries should take a lesson from the weakness of the legal system.

#### 3.1.2 Financial status of condominium

The condominium was constructed with a 7 percent interest loan from Commercial Bank of Ethiopia. The beneficiaries of condominiums built in 2007 and 2008 were integrated to Commercial Bank of Ethiopia Gondar Branch. As a result, the beneficiaries pay a monthly fixed amount of money to the Commercial Bank of Ethiopia based on their house typology. The cost of condominium houses per meter square (m^2^) based on year and housing typology are shown in Table 1.

**Table 1:** Financial cost of condominium houses.

S.N	Housing Typology	Cost per meter square			Payback year	
		Low (2007)	Highest (2008)	Average		
1.	Studio	1269.23	2487.14	1878.18	25	
2.	One-bedroom	1813.19	3553.05	2683.12	20	
3.	Two-bedroom	1903.85	3748.47	2826.16	20	
4.	Three-bedroom	1994.50	3801.76	2898.13	15/20	
5.	Commercial	1269.23	Varies	---	20	

Source: Commercial Bank of Ethiopia Gondar Branch and Gondar City Administration Housing Development and Administration Office (2020)

The cost per square meter varies depending on the housing typology. In both years, a three-bedroom house was relatively more expensive than other housing typologies. The payback term was between 15 and 25 years (see Table 1).

In the interview conducted, the loan return varied by location, with the rate of return in Azezo being lower than other locations due to poorer satisfaction with the quality of the buildings. One of the respondents responded like this:

“I have a condominium in Azezo site but the quality of the houses is poor and the cost of the loan is beyond the actual area, i.e., the amount requested to be paid is more than what it will be on the actual day. Thus, we were able to claim it, but I’m not satisfied with their response. Since I haven't had the option, I’m paying slowly with little money.”

Generally, the unit cost prices were varied in both years. The return rate was depended upon the quality and satisfaction of the beneficiaries.

#### 3.1.3 Condominium housing supply

The amount of housing available did not align with the demand for it. In developing country, such as Ethiopia, the proportionate difference is very significant. As shown in table seven, the total number of houses in Gondar was 74,333, whereas the entire condominium supply was 3346 housing units. Here, we can illustrate using simple arithmetic that the share is low, which is 4.5 percent. The details of condominium housing units are presented in figure two.

**Figure 2:**
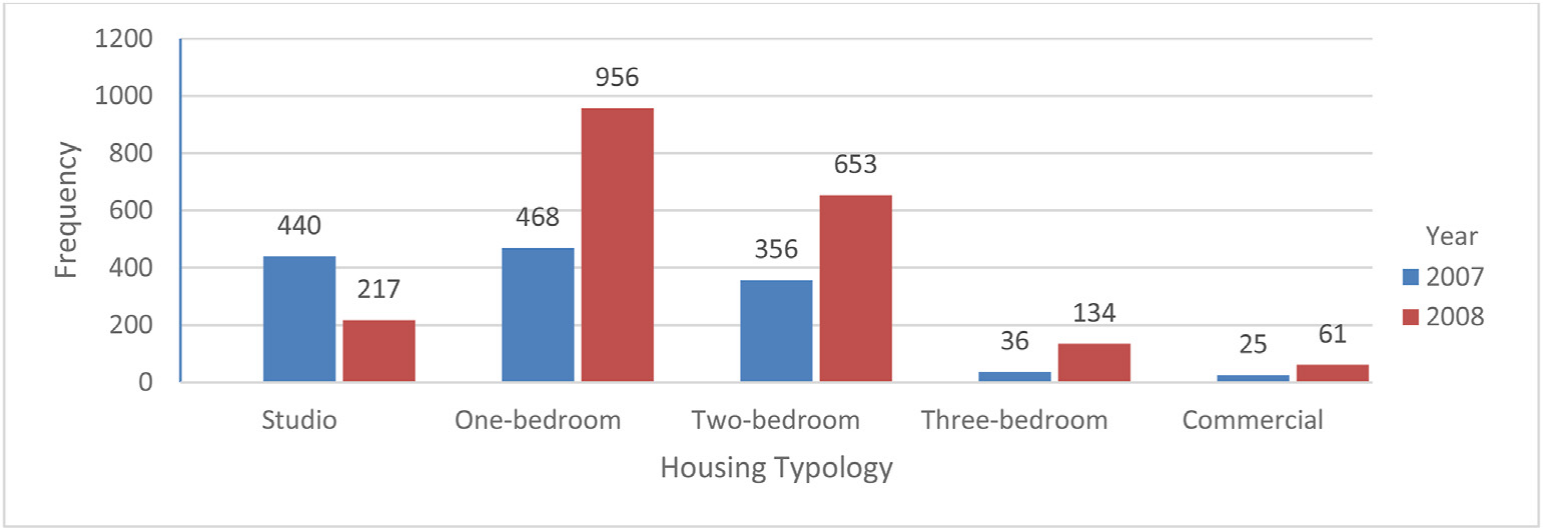
Supplied Condominium Housing units based on Housing Typology (source: Gondar City Administration Housing Development and Administration Office, 2020)

In both years, the one-bedroom housing type accounted for the highest percentage of total housing units (Figure 2). The three-bedroom housing type, on the other hand, was quite small next to commercial housing. In general, the supply of one-bedroom condominium housing was relatively high. However, the condominium houses in the city were often smaller than other housing types.

#### 3.1.4 Administration

The city administration built the condominium houses with a financial loan from the Commercial Bank of Ethiopia. After the construction was completed, the housing units were given to the beneficiary by entering into an agreement that incorporates the payment (amount of total, first instalment, and payback year), rights, and duties of the beneficiaries, as well as the loss of the agreement. The houses were transferred to the house beneficiaries once they were furnished. The administrations were assigned to a hypothetical housing unit association by the city government's housing development and administration office.

**Figure 3:**
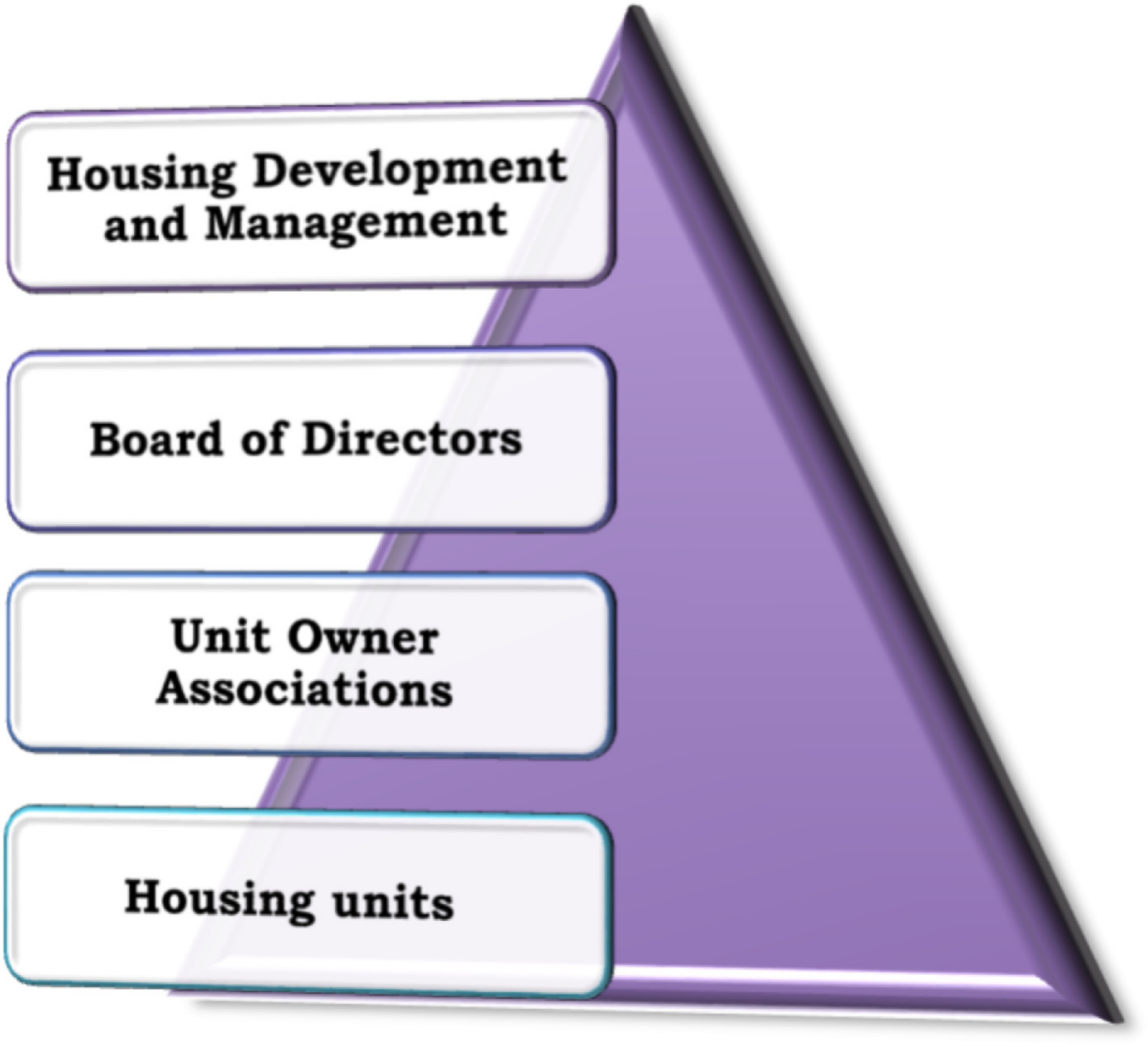
Administration hierarchical model of condominium house (source: developed by the Author, 2020)

After conducting interviews and document reviews, the administration's hierarchical model is shown in Figure 3.

According to Proclamation No. 370/2003, housing units were owned by individual homeowners who formed unit owner associations. The associations were organized by building, and the bylaws detailed their responsibilities. The housing unit is managed by a unit owners association that was formed with the goal of mutual benefit (Pro. 370/2003 Article 10 and 11 (1)). The association has the authority to fix the use of common elements and their upkeep (Article 27 (2)).

According to the interviews, several housing unit associations were not effective since the majority of the homeowners were renters. Regarding the administration, the interviewee responded that:

“The majority of the owners did not reside there. In our building, for example, out of 25 housing units, only five were owned by occupants; as a result, the associations were ineffective. Even though there are some committees, they are working voluntarily. Furthermore, the tasks and duties of housing management and administration were unclear.”

In general, the administrations of the condominium were not clearly specified, and some of the essential chores were done voluntarily by certain selected members.

### 3.2 Actors in condominium development

Condominium construction is a complex undertaking that necessitates the participation of several stakeholders at various levels. As presented in Table 2, the actors included government institutions, contractors, private companies, and financial institutions, including the Commercial Bank of Ethiopia.

**Table 2:** Actors and their roles in condominium houses.

Actors	Role of Actors
Commercial Bank of Ethiopia	• Borrowing money to the beneficiary and government.
Government institutions (kebele, construction and housing development agency)	• Providing identification card for the proofing of residents or not. • Checking whether beneficiary had house previously. • Creating linkage condominium beneficiary to the financial sectors.
Municipality	• Design the structural planning and follow up the construction progress. • Providing land for housing and infrastructures like road
Micro and Small Enterprising	• Organized construction cooperative and contractors. • Identify the community (youth) that would be involved in the construction for job opportunity.
Water Supply and Sewerage Service Office	• Providing water to the residents.
Ethiopian Electric Power Corporation	• Installation and delivering power to the condominium beneficiary.
Condominium Beneficiary	• Pay at least the first installment and fulfilled the required criteria like residential identification, proof that indicate homeless previously.
Contractors	• Construct as per the design and time frame.

Source: Interview conducted with key informants (2020).

As in the interview conducted about the actors, the respondents responded like this:

“The condominium program was designed to benefit residents that do not own a house in the city. The local government was responsible for selecting the beneficiary. However, due to a lack of supervision and control by the lower government in the selection of beneficiaries, some of the private house owners were incorporated as beneficiaries. The participation of the community in the screening of beneficiary and monitoring of the construction process was null. i.e., why misplaced benefiter allocation and the quality become low.”

Generally, the poor role implementation of the actor leads to poor quality of the building, resource exploitation, and limits the chances of others acquiring a home.

### 3.3 Role of condominium housing

Condominium houses play a variety of roles in reducing urban problems such as transportation provision costs, horizontal city expansion, farmer expropriation, reduced service costs, reduced degradation of environmental quality, improved water supply, reduced development of slums and informality, and housing problems. The following roles were identified based on data obtained from observations, interviews, reports, and satellite images evaluated using Arc GIS version 10.2.

#### 3.3.1 Land management

Land management is the process of putting land to good use, which may be true in having a diversified use of the land without wasting it. In order to determine whether land is managed or not, comparing the existing condominium area and the area of proposed private plot-based housing is an interesting issue.

Table 3 shows that the total area of the five condominium sites was 182,613.77 m^2^ with a total of 3346 households. The total area of the condominium buildings is 30,609m^2^ including the corridors, with the remaining 152,004.77m^2^ being road, community space, and drainage.

**Table 3:** The area covered with condominium and estimated private plot-based house.

House Type	Site Area	Buildings Area	Average Area of Road	Total Households
Condominium	182,613.77 m^2^	30,609 m^2^	35,126.62m^2^	3,346
Estimated Private plot-based house	>1,337,820m^2^	669,200m^2^	668,620m^2^	3,346

Source: Aerial photograph computed by Author (2020).

During the construction of the condominium, the private plot-based home permission was 200 m^2^. Thus, excluding drainage, roadways, and other public areas, the total private plot-based dwelling space for 3346 households would be 669,200 m^2^. The areas of the road are determined by the number of blocks, which is approximated using the following formula:

Let's say.

L = length of the block
W = Width of the block
S1 = width of the road excluding the width of the block
S2 = length of the road excluding the length of the block
n = number of the blocks
AB = area of the block
AR = total area of the Road
AR+B = area of block and road

(1) AB=L×W

(2) AR=AR+B−AB

$$\begin{array}{l} AR+B=\left(2S2+L\right)\left(2S1+W\right)\\ AR+B=4S2S1+2S2W+2S1L+LW\\ AR=4S2S1+2S2W+2S1L+LW-LW\\ AR=4S2S1+2S2W+2S1L \end{array}$$

For “n” number of block the area of the road using Eqs. (1) and (2) will be:

(3) AR(n+1)=(n+1)(4S2S1+2S1L+2S2W)−n(2S2S1+S1L)

When the blocks are place North to South and vise-verse

(4) AR(n+1)=(n+1)(4S2S1+2S1L+2S2W)−n(S2W+2S1S2)

When the blocks are place East to West, North to South and vise verse

(5) AR(n+1)=(n+1)(4S2S1+2S1L+2S2W)−n(S2W+2S1S2+S1L)−K

Where K is the conjunction of the road between more than three blocks.

K=(n−2)(S1S2) Where n is the number of blocks

Thus, the area of the road may be calculated using either of the above equations, depending on the orientation of the blocks. Road areas where the block orientation is either easting or northing can be determined using Eq. (4). When the blocks' geographical orientations are in all directions, Eq. (5) is applicable (east to west and north to south). The blocks were most likely built with equal delineation in all directions and the projected road areas for 3346 houses are presented in Table 4.

**Table 4:** Estimated Road area of plot-based houses.

Infrastructure Types	width of road (S1)	Length of Road (S2)	Width of block (W)	Length of block (L)	Housing per block	Average blocks (n)	Area of Road
Road	6–20m	8–30m	40m	180–240m	18–24	186–140	404,840m^2^−932,400m^2^

Source: projected by author (2020).

The width of the road within and across the block varied from six to twenty meters. The road's length ranged from eight to thirty meters. The existence of minimum and maximum road areas is caused by variations in length and width, as well as average blocks and house orientation (see Eqs. (3), (4), and (5)). As a result, the road area for 3346 plot-based houses ranged from 404,840m^2^ to 932,400m^2^. In general, with the implementation of the condominium house development program in the city of Gondar, almost 7.34 times the amount of urban land used for condominiums has been reclaimed. As a result of the constructed condominium, the total area to be transferred through private land-based land use would be reduced by more than 734 percent.

#### 3.3.2 Minimize cost of infrastructure

The cost of infrastructure covers the costs of road development, water supply, and power. Because condominium houses are growing vertically, the cost of providing roads, installing power, and supplying water within the block will be reduced. As it is shown in Table 3, the overall coverage of the condominium sites in Gondar, including common houses, roads, and open spaces, was 182,613.77m^2^.

Most cities grow in both directions. In this case, the area of the road would be determined using Eq. (5) i.e.

$AR(n+1)=\left(n+1\right)\left(4S2S1+2S1L+2S2W\right)-n\left(S2W+2S1S2+S1L\right)-K$ (see Table 4),

Here we had minimum and maximum area of the road and the total cost can be estimated using Eq. (6):

(6) Total cost = cost per m^2^ ∗Area of the road

The comparisons of the anticipated costs for road building for private plot-based houses and condominium houses using Eq. (6) are shown on Table 5.

**Table 5:** Cost of Road for plot-based private houses and condominium.

Infrastructure Types		Cost per m^2^	Area of Road		Total cost	
			Private land-based houses	Condominium	Private land-based houses	Condominium
Road	Cobble- Stone	810 birrs∗	404,840m^2^−932,400m^2^	35,126.62 m^2^	327,920,400 to 755,244,000 Birr	28,452,562.2 birr

Source: Satellite image and Gondar city Urban Development and Housing; infrastructure provision office (2021).

As shown in Table 5, the condominium's road area was 35,126.62m^2^ and the road area of plot-based houses ranged from 404,840 to 932,400 square meters. The total cost of cobblestone road construction for condominiums was less than 11.5 to 26.5 times that of a road for plot-based houses. Generally, condominiums reduce the required costs for road provision as compared to private plot-based houses.

#### 3.3.3 Improve affordability for housing

If a household engages in the construction of commonly (shared) houses, the cost of the household can be shared with their neighbours. However, the costs are prohibitively high when each household is required to have their own plot-based house (see Table 6).

**Table 6:** Cost comparison of private plot-based houses and condominium houses.

Types of building		Housing types			
		Plot-based house		Condominium	
		Minimum	Maximum	Minimum	Maximum
Cement houses	Cost per meter square	1269.23	3,801.76	1269.23	3,801.76
	Permitted Area	90	180	25.59	71.17
	Total Cost in birr	114,230.7	684,316.8	32,479.6	270,571.66

Source: Computes from the agreement of condominium owner, planning office and Commercial Bank of Ethiopia Gondar Branch (2020).

Table 6 shows that the minimum cost for constructing a plot-based house was 114,230.7 birr, but the minimum cost for constructing a condominium was 32,479.6 birr. For private plot-based houses and condominium houses, the maximum cost was 684,316.8 and 270,571.66 birr, respectively. In this case, the maximum cost of a plot-based house was raised by 253 percent, while the minimum cost of a plot-based house was 351.7 percent of the condominium.

In general, the cost of a plot-based house increased due to a difference in the total area permitted to be covered. The coverage areas of the condominiums were smaller than those of the private plot-based houses. Thus, the reduction in the total cost of housing construction due to the lower area makes the condominiums more affordable than plot-based houses.

#### 3.3.4 Minimize informality

The majority of informants existed primarily for the purpose of housing and speculation. As shown in Table 6, the cost of constructing a condominium was low, which discouraged the intention of involving informal land holding. Condominium construction occurred in 2007 and 2008, but the programs were not sustained. Because of the program stack, as shown in Table 7, informal houses in the city shared 17.4 percent, which was higher than condominium houses.

**Table 7:** Constructed houses in the city of Gondar since 2019.

House Type	Total Number	Share of Total House
Private House	32,290	43.4
Informal House	13,000	17.4
Condominium	3,346	4.5
Cooperative House	4,756	6.5
Government	12,451	16.8
Public Private Partnerships	431	0.6
Real Estate Houses	230	0.3
Rental house	7,829	10.5
Total	74,333	100

Source: Gondar City Administration Housing Development and Administration report (2020).

Regarding the expansion of informal, the interviewee responded as follows:

“During the commencement of the condominium, the intentions were to benefit the homeless urban dwellers from the program. In the city, the level of informality was low. But, after the wedge of the program, most of the residents intended to hold the plot informally.”

Many residents were intended to participate in informal activities due to stacked condominium housing development programs, high demand for housing, and a low ability to participate in the formal land holding system.

#### 3.3.5 Reduced housing shortage

In the country, there are various housing types, one of which is the condominium. According to the Gondar city administration housing development and administration office, there were 74,333 houses of various types in 2019.

As shown in Table 7, the share of condominiums in total houses was low, accounting for 4.5 percent, which was insignificant when compared to plot-based houses. In general, the condominium houses in the study area were limited. Despite this, the roles for accessed people played a larger role in reducing housing problems.

#### 3.3.6 Reduced expropriation and compensation

Farmers and poor urban residents are facing difficulties as the government continues to displace them by taking over their land to meet the increasing demand for housing and public use. Despite being compensated, farmers in peri-urban areas were losing farmland at an alarming rate. According to the observations made in the study area, the urban fringes were included as part of the urban, and farmers were expropriated. They were becoming increasingly marginalized and destitute as their compensation package was meagre and they struggled to adapt to urban life and find work (Wubneh, 2018).

The condominium houses many people in one story and has a smaller physical expansion than land-based houses. As a result, the possibility of expropriation is reduced.

In the study area, if each of the 3346 condominium housing units had their own private plot-based house, the total area of the houses would be 669,200m^2^, and the average area of roads would be 668,620m^2^. The condominium, on the other hand, had an area of 182,613.77m^2^. Thus, it is estimated that more than 115.5 ha of land would be expropriated for housing and roads, in addition to being expropriated for condominium houses when the houses were built on a private plot of land.

According to Proclamation 455/2005, the land compensation fee for the expropriated has three major components, such as crop cost, displacement cost, and permanent improvement cost (Federal Democratic Republic of Ethiopia, 2003b). The following illustration, using an acre of land, shows the compensation estimates for plot-based houses:

(7) Total compensation for product = Area of the land ∗ estimated productivity of the land ∗ Average current price of the product ∗10 year

The area is the total area to be expropriated within the actual measured land area. The agricultural office determined the estimated productivity, which is based on the yield of the land per year, which can vary from year to year. The average current price was determined by the trade office, which estimated the yearly average price of the product item in the year. The price of the product varies depending on the type of product, but cereal products like teff, barley, and sorghum were common in the study areas. The averages of the dominant products were used to calculate the productivity of the land and the price of the product. The total area of the condominium and private plot-based houses was 18.26 ha and more than 133.78 ha, respectively (see Table 3). As a result, we can use the above formula to calculate the compensation (see equation 7). For example, if this occurred in 2018, the government expected to pay 54, 241, 101 birrs for compensation of expropriated farmers in order to provide plot-based housing.

Table 8 shows that the average price of the product data was not available prior to 2017, and there was no office that ran the evaluation, but it was run by officials organized into committees from various offices. However, the study used a minimum of 2018 and a maximum of 2020. According to these figures, the compensation for expropriated plot-based houses increased by 733 percent in both 2018 and 2020 compared to condominiums. As a result of the area disparity, compensation is needed more for plot-based houses than for condominium houses.

**Table 8:** Compensation estimation for private plot-based houses and condominium.

Year	Types of product	Estimated productivity per hectare	Average price of the product	Compensation for condominium expropriated	Compensation for plot-based houses
2012	Cereal	1694 k.g	Not Available		
2013	Cereal	1767 k.g	Not Available		
2014	Cereal	1825 k.g	Not Available		
2015	Cereal	1854 k.g	Not Available		
2016	Cereal	1967 k.g	Not Available		
2017	Cereal	3400 k.g	Not Available	-	
2018	”	3604 k.g	11.25 birr	7,403,517 birrs	54,241,101 birrs
2019	”	3347 k.g	24.64 birr	15,059,036.61 birr	110,328,473 birrs
2020	”	2900 k.g	37.67 birr	19,947,771.8 birr	146,145,285.4 birr

Source: Computed by Authors using Central Gondar Agricultural office and CSA agricultural sample survey data (2020).

In addition to the aforementioned compensation, there is also compensation for remnant products. That is, the aforementioned figure is not the only compensation amount.

## 4 Conclusion and recommendation

Condominium houses are managed and administrated under Proclamation 370/2003, which focuses on unit registration, certification, ownership, sale, and lease, as well as association and governance. Based on the proclamation, the regional states of Amhara enacted regulation 01/2017, which directed and administrated the condominium. In the study area, the absence of a penalty for improper implementation, comprehensiveness of the law, weak controls, and poor institutional support were identified in this regulation.

The Commercial Bank of Ethiopia provided funding for the condominium housing development program. The loan was provided for a variety of housing typologies totalling 3346 housing units, with repayment terms ranging from 15 to 25 years. The total number of condominium houses supplied was 3346, with the majority being one-bedroom units, and the loan return rate varied depending upon the quality of the building.

The condominium administrations did not follow the directives. The housing unit associations served as the administration's foundation and were in charge of determining the common elements and maintenance. The overall administration was poor due to a lack of follow-up, and the majority of residents were renters. The organization was ineffective. The committees worked for no salary or allowance, causing the administration to be ineffective.

Commercial Bank of Ethiopia, government institutions (kebele and construction and housing development agency), municipalities, small and micro enterprises, water supply and sewerage service offices, Ethiopia Electric Power Corporation, households, and contractors were among the participants in the integrated housing development programs. The study areas' contractors were private, and quality and transparency in all aspects necessitated public-private collaboration.

The condominium house plays a role in reducing urban problems. The city saved more than 1,337,820m^2^ of land in the study areas from 3346 households. The condominium implementation results in the reclamation of more than 7.34 percent of urban land, excluding open space in plot-based houses. The cost of road infrastructure was reduced by 11.5–26.5 times as a result of condominium houses rather than plot-based houses. Because of the low area coverage of condominiums, which leads to lower construction costs, homeowners can access their housing. Furthermore, people can afford condominiums rather than formally participating in the lease system. However, due to the program's inability to be sustained, the residents were redirected to informal housing. Despite the fact that the shares were low, the condominium helped to alleviate housing shortages. and other urban problems include the expropriation of residents in the buffer zone and surrounding farmers. As a result of the vertical development of the condominium story and its low coverage, the number of expropriated farmers and the city's compensation costs were reduced.

The researcher recommended the following points to the government body based on the data gathered and interpretation.

• The condominium proclamation and regulations have to be revised.
• Developed institutional frameworks for associations with clear rules and regulations and recruited administrators to control and effectively manage them.
• Encourage other private banks to participate in the borrowing of money for housing as a social obligation mortgage.
• Create strict condominium regulations and an administration system.
• Plan for the continuation of condominium construction in order to increase housing supply, reduce expropriation and construction costs, infrastructure, informality, and improve housing affordability.

## Declarations

### Author contribution statement

Bantayehu Ayalew Workineh: Conceived and designed the experiments; Performed the experiments; Analyzed and interpreted the data; Contributed reagents, materials, analysis tools or data; Wrote the paper.

### Funding statement

This research did not receive any specific grant from funding agencies in the public, commercial, or not-for-profit sectors.

### Data availability statement

The data that has been used is confidential.

### Declaration of interest's statement

The authors declare no conflict of interest.

### Additional information

No additional information is available for this paper.

## Appendix A Supplementary data

Suplementary map.docx Suplementary map.docx
