# Supplementary material for: The practice and roles of condominium housing for tackling urban problems in the case of Gondar city, Ethiopia
Source: Heliyon. 2022 Dec 2;8(12):e11957. doi: 10.1016/j.heliyon.2022.e11957 (PMC9732299; doi:10.1016/j.heliyon.2022.e11957)
Supplement: Suplementary map.docx [file mmc1.docx]

There are five condominium site in the Gondar city administrataion. The research were undertake in these site. Each of the locational map of the study sites is presented below as supplementary material.

Figure 1.1: Map of the study site (source, Ethiopian mapping agency 2005: modified by author)

Figure 1.2: Map of the study site (source, Ethiopian mapping agency 2005: modified by author)

Figure 1.3: Map of the study site (source, Ethiopian mapping agency 2005: modified by author)

Figure 1.4: Map of the study site (source, Ethiopian mapping agency 2005: modified by author)

Figure 1.5: Map of the study site (source, Ethiopian mapping agency 2005: modified by author)
